# Proportions and risk factors of chronic obstructive pulmonary disease and preserved ratio impaired spirometry, and association with small airway disease, in the positive screening older population from China: a cross-sectional study

**DOI:** 10.1186/s12890-024-02920-2

**Published:** 2024-03-05

**Authors:** Le Sang, Xia Gong, Yunlei Huang, Jian Sun

**Affiliations:** 1 Shaoxing University, Shaoxing City, Zhejiang Province China; 2 Shaoxing People’s Hospital, Zhejiang Province Shaoxing City, China

**Keywords:** COPD, PRISm, SAD, Proportions, Risk factors, Early diagnosis

## Abstract

**Background:**

Early diagnosing Chronic Obstructive Pulmonary Disease (COPD) is relatively difficult. Therefore, the concepts of preserved ratio impaired spirometry (PRISm) and small airway disease (SAD) were proposed to achieve early diagnosis for COPD. Besides, the occurrence of COPD is positively related to age. However, the relationship among COPD, PRISm, and SAD still requires clarification. Thus, we estimated the proportions and risk factors of COPD and PRISm in the positive screening participants, and searched the methods of early diagnosing COPD via the SAD indicators.

**Methods:**

A total of 53,641 residents aged more than 60 years old from Shaoxing City, Zhejiang Province, China, completed a series of screening projects. And 2327 of positive screening participants ultimately finished bronchodilator tests. The data were statistically analyzed to figure out the proportions and risk factors of COPD and PRISm, and the efficacy of early diagnosing COPD by the SAD indicators.

**Results:**

Totally 2229 positive screening participants were included, the proportion of PRISm was 6.3% (141/2229), and of COPD was 78.2% (1743/2229). Statistical analyses showed that COPD patients were more likely to be smokers, males, and older. And COPD patients had higher questionnaire scores, meaning that they were more prone to have family history of respiratory diseases and more severe respiratory symptoms. Additionally, COPD patients had lower maximal mid-expiratory flow (MMEF) pred, forced expiratory flow (FEF) 75pred, and FEF50pred. And we found that male sex and presence of respiratory symptoms might lead to COPD and PRISm. Also, the methods of early diagnosing COPD through the SAD indicators might be acceptable.

**Conclusion:**

There is a close association between COPD and decreased small airway function (SAF) among the participants included. Age, smoking, male sex, worse SAF, and respiratory symptoms might cause the progressing from normal people to PRISm, then to COPD patients. Besides, the SAD indicators such as MMEFpred, FEF75pred, and FEF50pred were included in lung function tests and bronchodilator tests. Intriguingly, it was found that early diagnosing COPD via the SAD indicators might be feasible. In the future, early diagnosis for COPD requires further research.

## Introduction

COPD is a common, preventable, and treatable disease characterized by persistent respiratory symptoms and airflow limitations, typically associated with airway and (or) alveolar abnormalities caused by significant exposure to harmful particles or gases. Nowadays, with the development of disease screening and health education, COPD has been known gradually. However, the proportion and severity of COPD are underestimated. The data show that by 2019, COPD had become the third most common cause of death worldwide [1], and the proportion of COPD in China increased by 67.8% in 2019 compared with the survey results in 1990 [2]. Besides, to an extent, COPD is almost incurable. And it is known that COPD patients with effective self-management strategies will suffer from less disease burden and have better life quality. Therefore, COPD should be taken seriously in China. Yet, early diagnosing COPD is relatively difficult due to patients’ lack of obvious respiratory symptoms, and there are quite low follow-up rates even in the positive patients screened via questionnaires and lung function tests. Accordingly, scholars proposed the concepts of “early COPD” or “pre-COPD” to achieve early screening and effective management of COPD [3, 4].

“pre-COPD” was proposed by Global Initiative for Chronic Obstructive Lung Disease (GOLD) in 2022 [5], which refers to patients of any age who currently have no airflow restriction, regardless of whether they have detectably structural or functional abnormalities or respiratory symptoms, and they might or might not eventually have persistent airflow restriction. But the definition of “pre-COPD” still requires to be more accurate. For instance, Cosío and colleagues [6] defined “pre-COPD” as presence of > 5% of emphysema and (or) bronchial thickening by computed chromatography (CT) scan and (or) diffusing capacity of the lung for carbon monoxide (D_LCO_) < 80% of predicted in subjects with respiratory symptoms and post-bronchodilator forced expiratory volume in 1 s/forced vital capacity (FEV1/FVC) > 70%. And they found that of the studied population, 22.3% could be diagnosed with “pre-COPD”.

PRISm is utilized for screening “pre-COPD”. Currently, the diagnostic criteria of PRISm are based on FEV1/FVC ≥ 70% and FEV1 < 80%. Notablely, the diagnosis of COPD and PRISm must be conducted after participants take bronchodilators. A cohort study showed that among subjects with PRISm, 22.2% transitioned to COPD in GOLD 0, and 25.1% transitioned to GOLD 1–4. Moreover, patients with PRISm had worse lung function and less optimistic prognosis [7], which suggests PRISm represents a transitional state before established COPD. A survey showed that the incidence of PRISm was 7.1–25.2% [8–10], and the patients with PRISm were more prone to be symptomatic and with higher questionnaire scores.

Meanwhile, SAD is also closely related to “pre-COPD”. In humans, lower airways which extend down from airways are classified into the 8th-25th generation (taking trachea as the 1st generation to alveoli as the 23rd). Small airways including bronchioles, terminal bronchioles, respiratory bronchioles, alveolar ducts, and alveolar sacs are usually defined as those of < 2 mm diameter and without cartilage, located approximately by the 8th generation [11]. The cilia swing frequency of small airways is lower than that of large airways, and small airways lack progenitor basal cells. Therefore, harmful particles generated by cigarette combustion or atmospheric pollution are more likely to deposit on the surface of small airways [12]. Currently, the diagnostic criteria of SAD are on the basis of at least two of the following three indicators of lung function that are less than 65% of predicted: MMEF, FEF50%, and FEF75%. A sampling survey conducted in more than 10 provinces in China showed that the proportion of SAD was 43.5%, and SAD patients had higher mean age [13]. A longitudinal study of a COPD cohort also showed that small airways’ changes preceded large airways’ abnormalities [14]. Therefore, it is worth clarifying the relationship among COPD, PRISm, and SAD in elderly people.

To figure out the proportions and risk factors for COPD and PRISm in positive screening residents, and identify the effective methods of early diagnosing COPD by the SAD indicators, we screened the old residents in Shaoxing City, Zhejiang Province, China. And via analyzing the data, the demographic characteristics, living behaviors, respiratory symptoms, and SAF were compared among different groups divided by lung function.

## Materials and methods

### Participants

From 1st May, 2022 to 31st March, 2023, 2327 elderly residents living in Shaoxing City, Zhejiang Province, China, were selected. Individuals were excluded if data were lost or erroneous.

**Fig. 1:**
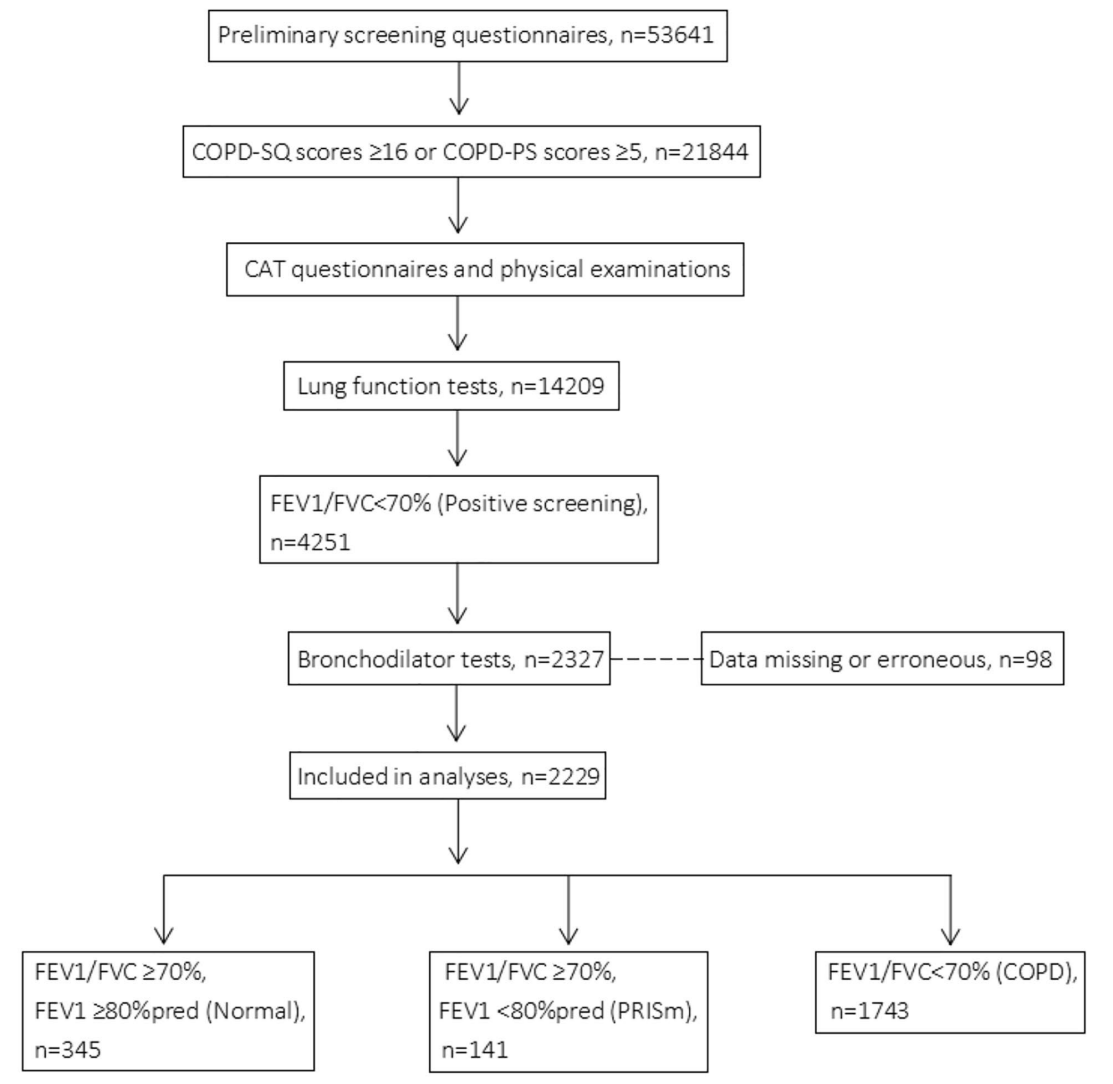
Study flow chart. Abbreviations: COPD:Chronic obstructive pulmonary disease; PRISmPreserved ratio impaired spirometry; COPD-SQ:COPD screening questionnaire; CAT:COPD assessment test; FEV1/FVC:Forced expiratory volume in 1 s/forced vital capacity

A total of 53,641 participants completed preliminary screening questionnaires, and 47% (21,844/53,641) of participants finished subsequent baseline questionnaires and physical examinations. Then 65.0% (14,209/21,844) of participants completed lung function tests, of which results showed that 29.9% (4251/14,209) had FEV1/FVC < 70%. Finally, 54.7% (2327/4251) of participants completed bronchodilator tests, while only 6.5% (152/2327) of patients finished follow-ups (Fig. 1).

### Preliminary screening questionnaire investigations

Participants were screened via COPD Screening Questionnaire (COPD-SQ) and COPD Population Screener Questionnaire (COPD-PS), of which contents contained demographic characteristics, living behaviors, and respiratory symptoms.

Residents finished questionnaires on Qiyi application (APP) after their ID cards were scanned at local primary health centers. There were a total of 76 primary health centers from 6 districts of Shaoxing City in the study.

### Baseline questionnaire investigations and physical examinations

Those participants with COPD-SQ scores ≥ 16 or COPD-PS scores ≥ 5 were asked to complete further baseline questionnaires such as COPD Assessment Test (CAT) and physical examinations at designated institutions. The data of questionnaire investigations and physical examinations were subsequently analyzed.

### Lung function tests and bronchodilator tests

Trained operators conducted lung function tests using spirometers such as “UBREATH”, “BreathHome,” and “XEEK”. Partial spirometers can connect to Qiyi APP to input the data automatically. Otherwise, operators would input the data manually. Those participants with FEV1/FVC < 70% in lung function tests were identified as the positive screening population, in whom subsequent bronchodilator tests were conducted. Dilators such as Salbutamol Sulfate Aerosol (Ventolin) were used to dilate bronchus in the short term, and bronchodilator tests were done after 15 min.

Individuals with COPD-SQ scores ≥ 16 and (or) COPD-PS scores ≥ 5, as well as those with FEV1/FVC ≥ 70% and (or) post-bronchodilator FEV1/FVC ≥ 70%, were defined as the population at high risk for COPD. Individuals with COPD-SQ scores ≥ 16 and (or) COPD-PS scores ≥ 5, as well as those with FEV1/FVC and post-bronchodilator FEV1/FVC ≤ 70% were defined as the population of suspected COPD. According to the GOLD 2023, all participants with an increase in FEV1 > 12% and > 200 ml in bronchodilator tests were defined as positive bronchodilator responses. Then participants positive in bronchodilator tests and those with suspected COPD were asked to complete follow-ups.

### Statistical analyses

SPSS v.26.0 (IBM) was used for statistical analyses, and figures were drawn by Origin v.2021, SPSS v.26.0 (IBM), and WPS v.11.1.0.14309. Continuous variables were reported as means and median lines, and compared by the non-parametric test due to non-normal distribution. Categorical variables were compared using the Chi-square test. Also, the multivariate logistic regression analysis was performed to evaluate the influences of different variables for COPD and PRISm, and 95% confidence intervals (95%CI) were estimated. The variables related to COPD and PRISm with *p*-value < 0.2 in the univariate analysis were entered. The generalized linear mode was performed to assess the impact factors of COPD and PRISm. A two-tailed *p*-value of < 0.05 was considered statistically significant.

## Results

### Questionnaire investigations among normal, PRISM, and COPD participants

**Fig. 2:**
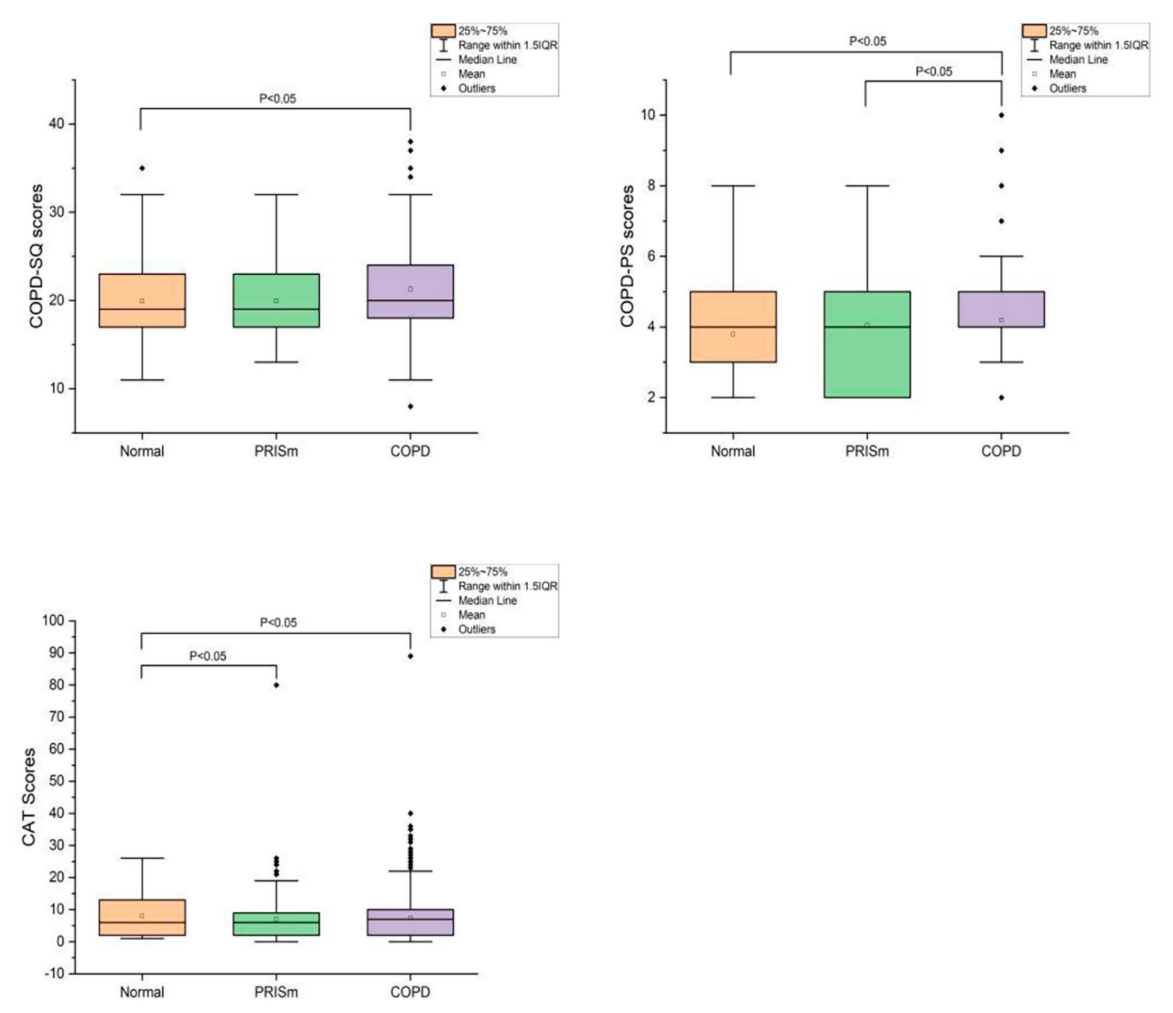
Questionnaire scores of participants. Abbreviations: COPD:Chronic obstructive pulmonary disease; PRISm:Preserved ratio impaired spirometry; COPD-SQ:COPD screening questionnaire; COPD-PS:COPD population screener; CAT:COPD assessment test

The questionnaire scores of the participants included were statistically analyzed, as shown in Fig. 2. Significant differences in COPD-SQ, COPD-PS, and CAT scores were found among three groups of participants. According to the results, the COPD group generally had higher questionnaire scores, meaning there were more severe symptoms and worse basic situations in COPD patients.

### Demographic characteristics among normal, PRISM, and COPD participants

A total of 2327 positive screening participants completed bronchodilator tests, and the data of 2229 were analyzed. The proportion of PRISm was 6.3% (141/2229), and of COPD was 78.2% (1743/2229).

In the study, we calculated Body Mass Index (BMI) as weight (kg) divided by height (m)^2^, and we defined those with BMI≥28.0 kg/m^2^ as obese patients. Among the total residents included, males accounted for 76.5% (1706/2229), 6.2% (138/2229) of participants had BMI≥28.0 kg/m^2^, and 24.4% (543/2229) had more than 90 cm waistlines. Additionally, 23.3% (519/2229) had family history of chronic respiratory diseases, while only 0.4% (10/2229) of participants had educational experience of more than 9 years.

Through the non-parametric test and the Chi-square test, it showed that there were significant differences in age, gender, BMI, and waistlines among three groups of participants. According to the results, COPD and PRISm patients were more likely to be males and older, and PRISm patients might have higher BMI and longer waistlines. Yet, there were no significant differences in educational background and family history (all *P* > 0.05). The demographic characteristics of all participants are shown in Table 1.

**Table 1:** Demographic characteristics of participants

	Total	Normal	PRISm	COPD	*P*-Value
**N**	2229	345 (15.5%)	141 (6.3%)	1743 (78.2%)	
**Age**					< 0.05
60–64	27 (1.2%)	7 (2.0%)	3 (2.1%)	17 (1.0%)	
65–69	1178 (52.8%)	176 (51.0%)	68 (48.2%)	715 (41.0%)	
≥ 70	1024 (46.0%)	162 (47.0%)	70 (49.7%)	1011 (58.0%)	
**Gender**					< 0.05
male	1706 (76.5%)	228 (66.1%)	96 (68.1%)	1382 (79.3%)	
female	523 (23.5%)	117 (33.9%)	45 (31.9%)	361 (20.7%)	
**BMI**					< 0.05
< 18.5	132 (6.0%)	19 (5.5%)	8 (5.7%)	105 (6.0%)	
18.5–23.9	1299 (58.3%)	195 (56.5%)	63 (44.7%)	1041 (59.7%)	
24–27.9	660 (29.5%)	111 (32.2%)	47 (33.3%)	502 (28.8%)	
≥ 28	138 (6.2%)	20 (5.8%)	23 (16.3%)	95 (5.5%)	
**Waistlines**					< 0.05
< 90	1686 (75.6%)	264 (76.5%)	97 (68.8%)	1325 (76.0%)	
90–99	438 (19.7%)	73 (21.2%)	29 (20.6%)	336 (19.3%)	
≥ 100	105 (4.7%)	8 (2.3%)	15 (10.6%)	82 (4.7%)	
**Education**					0.233
never	287 (12.9%)	43 (12.5%)	27 (19.1%)	217 (12.4%)	
< 9 years	1932 (86.7%)	301 (87.2%)	114 (80.9%)	1517(87.0%)	
≥ 9 years	10 (0.4%)	1 (0.3%)	0 (0.0%)	9 (0.6%)	
**Family History**					
yes	519 (23.3%)	72 (20.9%)	28 (19.9%)	419 (24.0%)	0.272
no	1710 (76.7%)	273 (79.1%)	113 (80.1%)	1324 (76.0%)	

### Living behaviors among normal, PRISM, and COPD participants

Regarding living behaviors, 33.9% (755/2229) of participants had never smoked, and approximately 33.7% (752/2229) were exposed to cooking smoke and biofuel. Also, 19.7% (440/2229) of participants were exposed to dust in the workplace, and only 9.8% (218/2229) had poor sleeping quality.

Through the Chi-square test, it was found that there were significant differences in smoking, cooking, and using coal or firewood among three groups of participants (Table 2). The results showed that COPD patients were more likely to be smokers, but both COPD and PRISm patients were less likely to be exposed to cooking smoke, which is contrary to our stereotype. And there were no significant differences in occupational dust and sleeping quality (all *P* > 0.05).

**Table 2:** Living behaviors of participants

	Total	Normal	PRISm	COPD	*P*-Value
**Smoking**					< 0.05
never	755 (33.9%)	154 (44.6%)	57 (40.4%)	544 (31.2%)	
previous	449 (20.1%)	62 (18.0%)	29 (20.6%)	358 (20.5%)	
current	1025 (46.0%)	129 (37.4%)	55 (39.0%)	841 (48.3%)	
**Cooking**					< 0.05
yes	752 (33.7%)	135 (39.1%)	41 (29.1%)	576 (33.0%)	
no	1477 (66.3%)	210 (60.9%)	100 (70.9%)	1167 (67.0%)	
**Using coal or firewood**					< 0.05
yes	754 (33.8%)	137 (39.1%)	41 (29.1%)	576 (33.0%)	
no	1475 (66.2%)	208 (60.9%)	100 (70.9%)	1167 (67.0%)	
**Occupational dust**					0.156
yes	440 (19.7%)	55 (15.9%)	29 (20.6%)	356 (20.4%)	
no	1789 (80.3%)	290 (84.1%)	112 (79.4%)	1387 (79.6%)	
**Sleeping quality**					0.802
good	2011 (90.2%)	311 (90.1%)	125 (88.7%)	1575 (90.4%)	
bad	218 (9.8%)	34 (9.9%)	16 (11.3%)	168 (9.6%)	

### Respiratory symptoms among normal, PRISM, and COPD participants

Respiratory symptoms among three groups were compared in the study. However, there were no significant differences in chronic cough, dyspnea, phlegm, and limited activities (all *P* > 0.05) (Table 3).

**Table 3:** Respiratory symptoms of participants

	Total	Normal	PRISm	COPD	*P*-Value
**Chronic cough**					0.107
yes	893 (40.1%)	121 (35.1%)	55 (39.0%)	717 (41.1%)	
no	1336 (59.9%)	224 (64.9%)	86 (61.0%)	1026 (58.9%)	
**Dyspnea**					0.141
yes	1392 (62.4%)	216 (62.6%)	99 (70.2%)	1077 (61.8%)	
no	837 (37.6%)	129 (37.4%)	42 (29.8%)	666 (38.2%)	
**Phlegm**					0.482
yes	1683 (75.5%)	268 (77.7%)	109 (77.3%)	1306 (75.0%)	
no	546 (25.5%)	77 (22.3%)	32 (22.7%)	437 (25.0%)	
**Limited activities**					0.168
yes	374 (16.8%)	46 (13.3%)	26 (18.4%)	302 (17.3%)	
no	1855 (83.2%)	299 (86.7%)	115 (81.6%)	1441 (82.7%)	

### Bronchodilator tests among normal, PRISM, and COPD participants

**Fig. 3:**
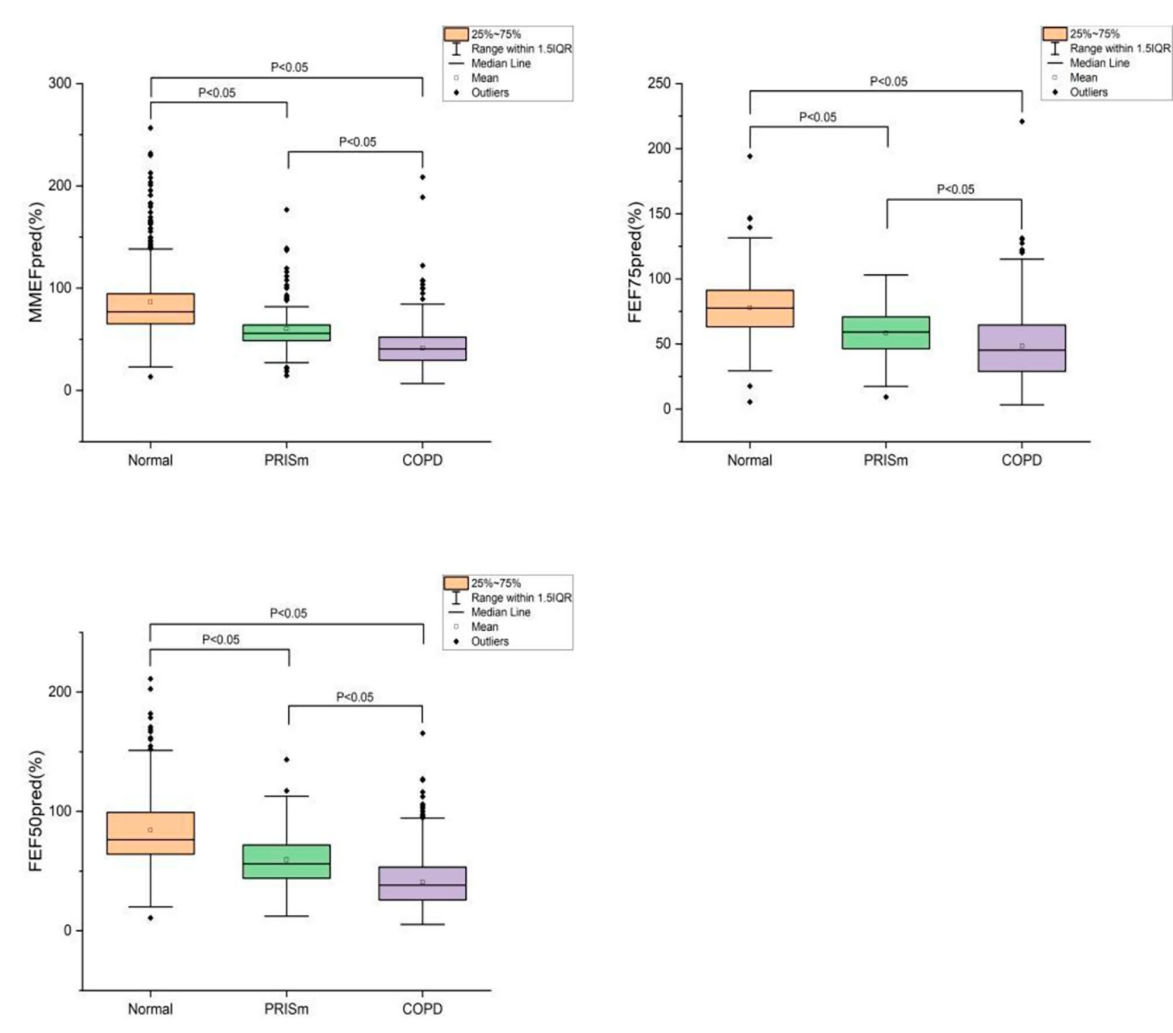
SAF of participants. Abbreviations: COPD:Chronic obstructive pulmonary disease; PRISm:Preserved ratio impaired spirometry; MMEF:Maximal mid-expiratory flow; FEF:Forced expiratory flow

The participants with FEV1/FVC < 70% in lung function tests were asked to finish bronchodilator tests. Three SAD indicators were included in the study, namely MMEFpred, FEF75pred, and FEF50pred. Through the non-parametric test, we found that all three indicators among three groups of participants differ significantly (Fig. 3). As compared with normal participants, PRISm patients had lower MMEFpred (60.30 ± 23.32 vs. 86.58 ± 35.39), FEF75pred (58.41 ± 18.94 vs. 77.712 ± 2.76), and FEF50pred (59.46 ± 21.83 vs. 84.45 ± 29.78). And as compared with PRISm patients, COPD patients also had lower MMEFpred (41.53 ± 16.81 vs. 60.30 ± 23.32), FEF75pred (48.18 ± 24.37 vs. 58.41 ± 18.94), and FEF50pred (40.56 ± 19.13 vs. 59.46 ± 21.83), which suggested that the SAF of the normal, PRISm, and COPD groups decreased gradually.

### The multivariate analysis of the data included

**Fig. 4:**
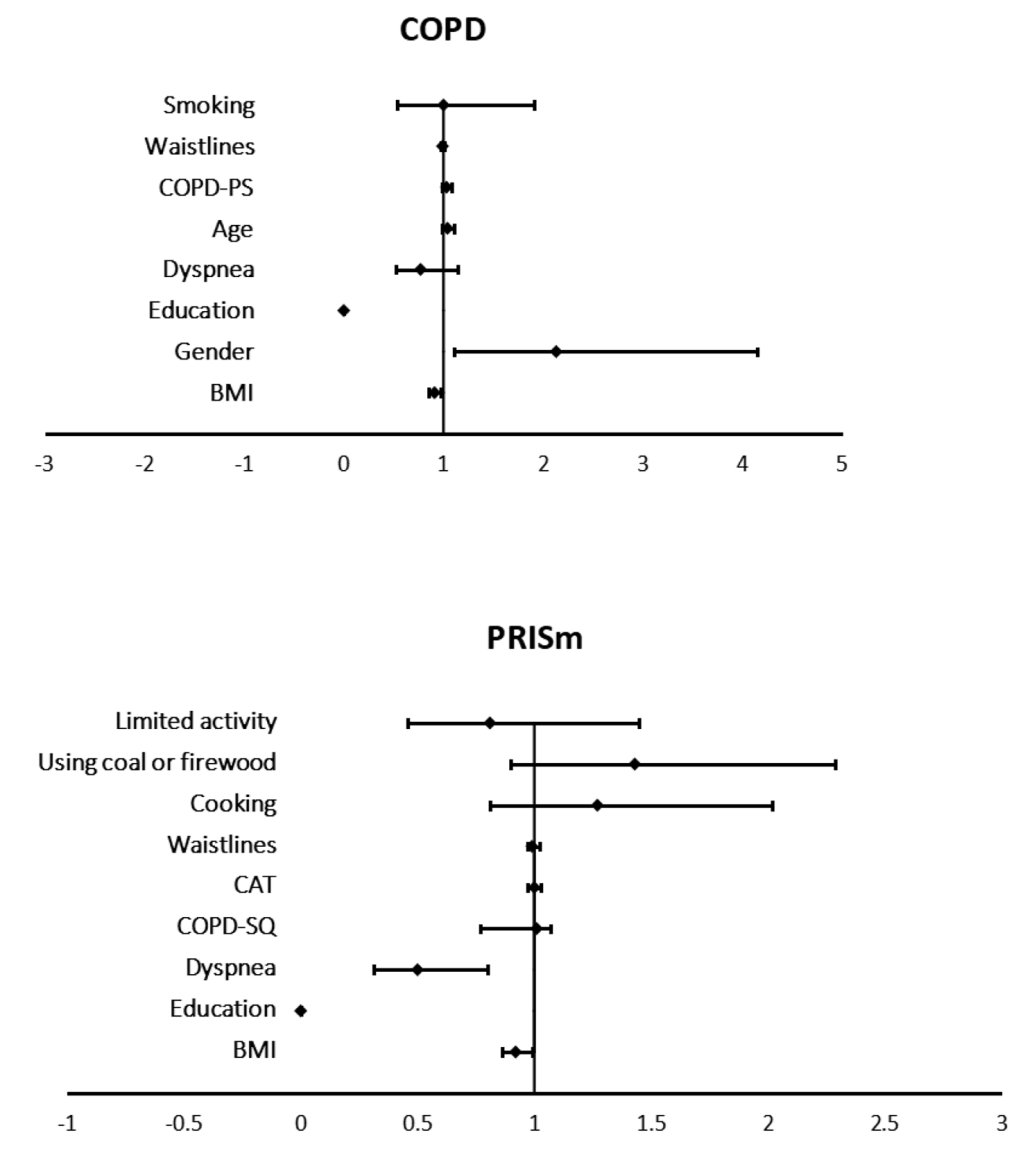
Impact factors related to COPD and PRISm. Abbreviations: COPD:Chronic obstructive pulmonary disease; PRISm:Preserved ratio impaired spirometry; BMI:Body mass index; COPD-SQ:COPD screening questionnaire; CAT:COPD assessment test

The variables with *p*-value < 0.2 in the univariate analysis were entered to be conducted the multivariate analysis. In the multivariate logistic model, BMI, educational background, and dyspnea were significantly associated with the progressing from normal population to PRISm patients (Table 4). And BMI, gender, and educational background were significantly associated with the progression from PRISm patients to COPD patients (Table 5) (Fig. 4). According to the results, male sex and presence of respiratory symptoms might lead to the progression from normal participants to PRISm, then to COPD patients. Yet, the impacts of indicators such as BMI and educational background still require to be further clarified.

**Table 4:** The multivariate analysis of impact factors related to PRISm

Normal	*P*-Value	β	EXP (β)	95%CI
**BMI**	< 0.05	-0.08	0.92	(0.86, 0.99)
**Education**				
never	< 0.05	-12.50	3.72E-6	(1.96E-7, 7.05E-5)
≤ 9 years	< 0.05	-12.46	3.87E-6	(2.15E-7, 6.96E-5)
≥ 9 years	/	/	/	/
**Dyspnea**				
yes	< 0.05	-0.69	0.50	(0.31, 0.80)
no	/	/	/	/
**COPD-SQ**	0.25	-0.10	0.91	(0.77, 1.07)
**CAT**	0.90	0.00	1.00	(0.97, 1.03)
**Waistlines**	0.51	-0.01	0.99	(0.97, 1.02)
**Cooking**				
yes	0.30	0.24	1.27	(0.81, 2.02)
no	/	/	/	/
**Using coal or firewood**				
yes	0.13	0.36	1.43	(0.90, 2.29)
no	/	/	/	/
**Limited activity**				
yes	0.49	-0.21	0.81	(0.46, 1.45)
no	/	/	/	/

**Table 5:** The multivariate analysis of impact factors related to COPD

COPD	*P*-Value	β	EXP (β)	95%CI
**BMI**	< 0.05	-0.10	0.91	(0.85, 0.97)
**Gender**				
male	< 0.05	0.76	2.13	(1.10, 4.14)
female	/	/	/	/
**Education**				
never	< 0.05	-13.50	1.378E-6	(8.43E-7, 2.25E-6)
≤ 9 years	< 0.05	-12.543	3.57E-6	(3.57E-6, 3.57E-6)
≥ 9 years	/	/	/	/
**Dyspnea**				
yes	0.19	-0.26	0.77	(0.52, 1.14)
no	/	/	/	/
**Age**	0.18	0.04	1.04	(0.98, 1.11)
**COPD-PS**	0.29	0.03	1.03	(0.98, 1.08)
**Waistlines**	0.28	-0.01	0.99	(0.97, 1.01)
**Smoking**				
never	1.00	0.00	1.00	(0.53, 1.91)
previous	0.17	-0.35	0.71	(0.43, 1.16)
current	/	/	/	/

### Diagnostic efficacy of MMEFpred, FEF75pred, and FEF50pred for COPD and PRISm

**Fig. 5:**
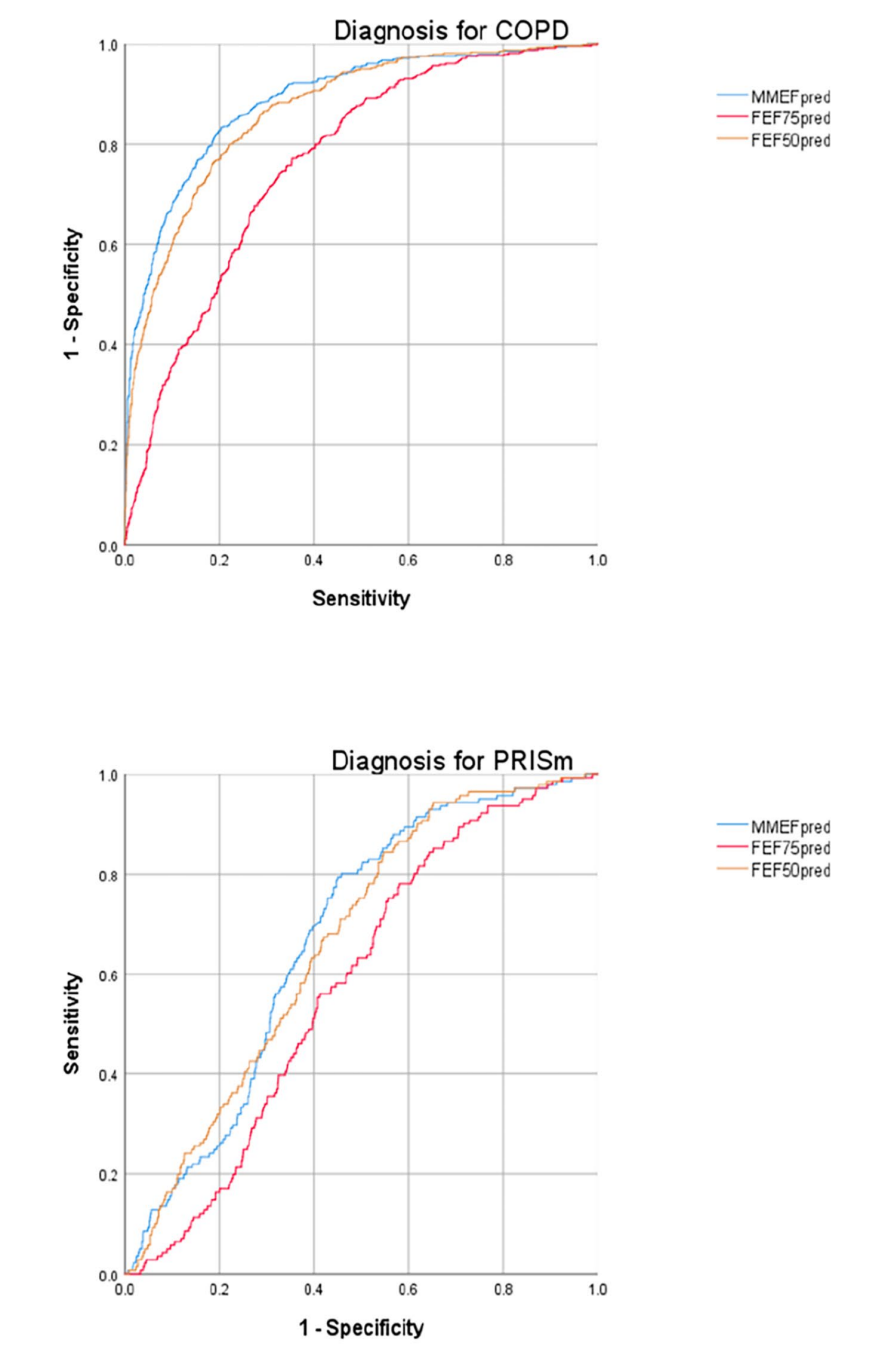
ROC curves of diagnosing COPD and PRISm. Abbreviations: COPD:Chronic obstructive pulmonary disease; PRISm:Preserved ratio impaired spirometry; MMEF:Maximal mid-expiratory flow; FEF:Forced expiratory flow

Based on the results above, it had been identified that lower MMEFpred, FEF75pred, and FEF50pred were associated with COPD and PRISm. Therefore, diagnosing COPD and PRISm through MMEFpred, FEF75pred, and FEF50pred is worth studying. Accordingly, the receiver operating characteristic (ROC) curve was drawn, and the area under the curve (AUC) and cutoff values were calculated (Fig. 5).

The results showed that the AUC of MMEFpred, FEF75pred, and FEF50pred was 88.70%, 76.50%, and 86.80%, respectively. And the cutoff values were 54.31% (sensitivity: 79.6%, specificity: 83.3%), 55.31% (sensitivity: 64.6%, specificity: 77.2%), and 55.45% (sensitivity: 77.9%, specificity: 80.0%), respectively. Besides, the AUC of the combined diagnosis with three indicators was 89.70% (sensitivity: 79.6%, specificity: 83.1%).

However, the AUC for PRISm was relatively low, which was 67.10%, 57.90%, and 66.20%, respectively. And the cutoff values were 34.5% (sensitivity: 79.4%, specificity: 55.1%), 5.76% (sensitivity: 99.8%, specificity: 0.2%), and 29.8% (sensitivity: 84.4%, specificity: 45.4%), respectively. The AUC of the combined diagnosis with three indicators was 66.60% (sensitivity: 92.9%, specificity: 30.1%).

## Discussion

The global proportion of COPD is constantly increasing, putting a great burden on patients and society due to its high morbidity and mortality [5, 15, 16]. Apart from COPD being largely underestimated and underdiagnosed [17], patients diagnosed with COPD are difficult to be cured thoroughly. And the disease will progress if risk factors exist constantly, bringing enormous challenges for clinical work. Obviously, the early diagnosis and prevention for COPD is urgent. Currently, an increasing amount of scholars are exploring “pre-COPD” [3], and PRISm and SAD are identified as transitional stages before COPD is diagnosed. Therefore, the data of 2229 positive screening participants were collected and analyzed in the study, expecting to estimate the proportions and risk factors for COPD and PRISm, and search the methods of early diagnosing COPD through the SAD indicators.

Among 2229 participants, COPD patients had higher COPD-SQ, COPD-PS, and CAT scores as compared with the normal and PRISm groups. Generally, the scores of questionnaires are positively related to the severity of diseases, meaning that COPD patients were more likely to have respiratory symptoms, which is accorded with the previous study [16]. Besides, seemingly distributing questionnaires might be an effective way for screening “pre-COPD”. Encouragingly, the popularization of electronic questionnaires lessens the cost of distributing questionnaires, promoting the utilization of such a method.

Additionally, there were higher proportions of males and the elderly in COPD patients, which is compatible with the previous findings [16]. The result suggested that older age and male sex might lead to the occurrence of COPD, verifying the necessity of conducting primary screening in older participants. BMI and waistlines are two risk factors with high relevance, representing participants’ body fat percentage. A previous study showed that higher BMI was a remarkable preventable risk factor for COPD [13]. However, in the study PRISm patients were found to have higher BMI and longer waistlines, indicating that BMI and waistlines might not be totally positively related to the severity of the disease. Also, educational background and family history did not differ significantly. Since the participants were the elderly whose education levels were generally low, thus the data had certain limitations. And it requires more sufficient data to determine whether COPD and PRISm were genetic diseases.

The data of living behaviors were also analyzed, showing that COPD and PRISm patients were more likely to be current and former smokers, enlightening that smoking might be a risk factor for COPD and PRISm. Notablely, the smoking rate is higher in males in China [18], which might explain the reality that males were more inclined to suffer from COPD. In terms of smoking, sadly, the smoking rate in China has increased in recent decades of years [13], warning us that advocating smoking cessation is necessary and urgent. Yet, we found that COPD and PRISm patients were less likely to be exposed to cooking smoke and biofuel, suggesting that the particles generated in daily life might not lead to COPD and PRISm. In contrast, a recent study showed that ambient air pollution exposure would increase the exosomes in residents’ serum. And it was believable that exosomes might have the potentials to induce inflammation via mechanisms such as the recruitment of neutrophils, which might partially explain the phenomenon that ambient air pollution exposure is significantly correlated to the occurrence of COPD [19]. In occupational dust and sleeping quality, no differences were found.

Besides, there were no significant differences in respiratory symptoms found in the study, which is consistent with the Canadian Cohort of Obstructive Lung Disease (CanCOLD) Study [20]. Considering that older participants originally had poor basic conditions, and they generally had respiratory symptoms under several specific situations such as where they had a common cold, which might interfere with the accuracy of the information obtained from questionnaires. More convincing data are required in further research.

In our study, the role and importance of the SAD indicators were emphasized. Therefore, we statistically analyzed the results of bronchodilator tests that the SAD indicators were included in. Intriguingly, the SAD indicators significantly differ between every two groups. Furthermore, it was shown that patients’ SAF decreased with the severity of diseases increased. Thus, it is reasonable to define SAD as “pre-COPD”. Given the situation where COPD, especially asymptomatic COPD is difficult to be early diagnosed, measuring participants’ SAD indicators can be an acceptable method of screening “pre-COPD”.

In addition, via the multivariate logistic regression, it was found that male sex and presence of respiratory symptoms might lead to the occurrence of COPD and PRISm. To an extent, the result was compatible with a Swiss study including the general population, which showed that individuals with respiratory symptoms such as cough, phlegm, or dyspnea had worse lung function [21]. Besides, we found that PRISm patients had lower education levels, which requires further study due to the forementioned data limitations.

Furthermore, we drew the ROC curve to determine whether diagnosing COPD and PRISm through the SAD indicators was feasible, expecting to figure out the methods of early diagnosing COPD. And the results showed that the efficacy of diagnosing COPD by SAF was acceptable, while for PRISm might be relatively unsatisfying. Besides, the thresholds of diagnosing were approximately 55%, with approximately 80% of sensitivities and specificities. Therefore, it is reasonable that early diagnosing COPD by the SAD indicators can be put into practice in the future.

Remarkably, only 152 patients with COPD completed subsequent follow-ups. Patients are unwilling to complete follow-ups might be because they are asymptomatic. Also, the self-funded examinations and medicine are unaffordable for partial residents, as well as most older participants lack the knowledge of COPD. Therefore, health education is urgent to be popularized. Fortunately, there has been a study of online diagnosis and visits, and encouraging results have been achieved. It showed that participants were more inclined to complete online follow-ups [22]. In the future, online diagnosis and treatments might be achieved in several advanced regions in China, expecting to acquire positive responses.

All participants were screened by clinical physicians and post-bronchodilator spirometry in the study. Nevertheless, the study also has some limitations. For instance, partial data might not be convincing enough because of the differences in operators’ skills in primary care centers, which is evidenced by the quality evaluation of lung function tests. Therefore, operators require to be further trained in the future. And because it is relatively difficult to control and monitor the extent of ambient air pollution in different districts of Shaoxing City. Unfortunately, the risk factor that ambient air pollution exposure was not included in the study. Additionally, the study can not assess long-term outcomes due to the limited rates of follow-ups.

## Conclusion

PRISm and SAD are identified as “pre-COPD”, of which diagnoses rely on SAF. In the study, positive screening participants completed bronchodilator tests. The results showed that MMEFpred, FEF75pred, and FEF50pred were related to COPD and PRISm, and were also associated with the progression of the diseases. Additionally, risk factors for COPD and PRISm such as age and male sex were verified statistically. Moreover, it was found that the efficacy of diagnosing COPD by MMEFpred, FEF75pred, and FEF50pred was acceptable, indicating the potentials of these indicators in early diagnosis for COPD. In the future, more effective and accurate methods of early diagnosing COPD require further research and verification based on more clinical data.

## Data Availability

The dataset used and/or analyzed during the current study are available from the corresponding author on reasonable request.

## Abbreviations

COPD: Chronic obstructive pulmonary disease
PRISm: Preserved ratio impaired spirometry
SAD: Small airway disease
SAF: Small airway function
GOLD: Global initiative for chronic obstructive lung disease
CT: Computed chromatography
DLCO: Diffusing capacity of the lung for carbon monoxide
FEV1/FVC: Forced expiratory volume in 1 s/forced vital capacity
MMEF: Maximal mid-expiratory flow
FEF: Forced expiratory flow
COPD-SQ: COPD screening questionnaire
COPD-PS: COPD population screener questionnaire
CAT: COPD assessment test
APP: Application
ROC: Receiver operating characteristic
AUC: Area under the curve
